# Exoscopic-Endoscopic Resection of Intramedullary Spinal Cord Metastasis From Renal Cell Carcinoma With Ventral Exophytic Extension

**DOI:** 10.7759/cureus.76362

**Published:** 2024-12-25

**Authors:** Hideki Hayashi, Hayato Nishikawa, Hirokuni Hashikata, Hiroki Toda

**Affiliations:** 1 Department of Neurosurgery, Medical Research Institute Kitano Hospital, PIIF Tazuke-Kofukai, Osaka, JPN

**Keywords:** cervical, endoscope, exoscope, intramedullary spinal cord metastasis, radiotherapy, renal cell carcinoma

## Abstract

Intramedullary spinal cord metastasis (ISCM) is a rare manifestation of renal cell carcinoma (RCC). A 73-year-old man presented with left shoulder pain and left upper extremity weakness for two months. Magnetic resonance imaging (MRI) revealed intramedullary and intradural extramedullary lesions at the C5 level, compressing the spinal cord from the center of the cord and the left ventral side. Contrast-enhanced CT revealed a right renal mass and brain MRI showed no other lesions. Digital subtraction angiography showed a tumor stain from the anterior spinal artery and subsequent angioarchitecture of the intra- and extramedullary tumors. Following exoscopic resection of the extramedullary tumor, the intramedullary tumor was removed via a posterior midline myelotomy. The tumor surrounding the anterior spinal artery was intentionally left to prevent neurological deficits. The histopathological examination revealed metastatic clear cell RCC. Postoperative MRI revealed a small residual tumor ventral to the spinal cord. The nephrectomy for the right RCC was performed one month after the initial spinal surgery. Within the subsequent one month, the residual tumor rapidly increased in size. Reoperation with exoscopic-endoscopic techniques achieved complete tumor resection. The patient underwent radiotherapy to the C3-6 levels (30 Gy in 10 fractions) and pembrolizumab therapy. Postoperative MRI demonstrated no recurrence for four months, and the patient’s symptoms remained in the same preoperative state. This case highlights the successful use of advanced minimally invasive techniques for treating ventrally exophytic ISCM from RCC.

## Introduction

Intramedullary spinal cord metastases (ISCMs) are an infrequent entity, constituting merely 0.1% to 2% of all spinal tumors, with the predominant origins being primary lung and breast malignancies [1,2]. Renal cell carcinoma (RCC), a highly vascularized tumor with a distinctive metastatic pattern, accounts for only 4-9% of ISCMs​ [3-6]. Notably, RCC exhibits a propensity for late-onset metastases, often manifesting several years or even decades following curative treatment of the primary tumor ​[1-3]. This protracted latency period underscores the necessity for long-term vigilance in RCC patients. ISCM pathological mechanisms are estimated to be arterial pathways assisted by the coexistence of lung and brain, the path through Batoson’s venous plexus, and direct invasion from the spinal extradural space, cerebrospinal fluid, or nerve roots [6].

The clinical presentation of ISCM is typically characterized by rapidly progressive neurological deficits, including motor weakness, sensory disturbances, and intractable pain, necessitating expeditious diagnostic and therapeutic intervention [1,3]. Despite advancements in imaging modalities, including contrast-enhanced gadolinium (Gd) magnetic resonance imaging (MRI), the differentiation between metastatic and primary spinal cord tumors remains diagnostically challenging due to overlapping radiological features​ [3].

Surgical intervention remains the principal therapeutic modality for ISCM, particularly in cases presenting with significant neurological compromise. However, the surgical management of ventrally located lesions poses formidable challenges due to restricted accessibility and the proximity of critical vascular structures such as the anterior spinal artery. While conventional surgical approaches often entail considerable risks to neurological function, minimally invasive techniques, including exoscopic and endoscopic methods, have emerged as promising alternatives. These techniques afford superior visualization and precision, enabling safer resection of lesions in anatomically constrained locations​ [7].

This paper aims to present a rare case of cervical ISCM with ventral exophytic extension from RCC, treated using a novel combination of exoscopic and endoscopic surgical approaches. The purpose of this study is to demonstrate the effectiveness and safety of these advanced minimally invasive techniques in achieving complete tumor resection while reducing the risk of iatrogenic neurological injury. By providing a detailed account of the surgical methods and outcomes, this report seeks to enrich the evolving discourse on ISCM management and offer practical guidance for addressing complex spinal cord lesions.

## Case presentation

**Figure 1:**
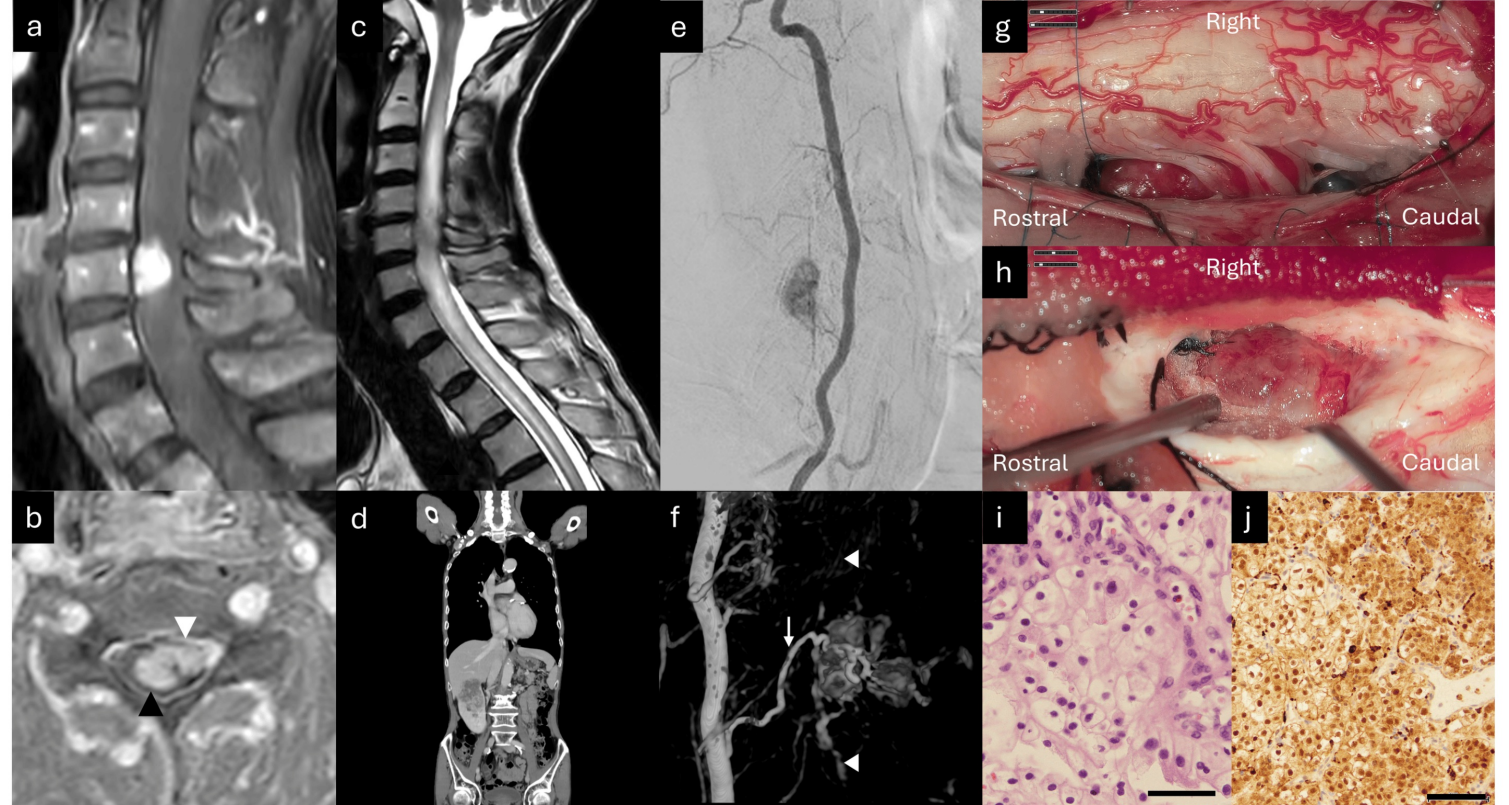
Preoperative and intraoperative findings during the first surgery (a) Initial sagittal contrast-enhanced gadolinium (Gd) magnetic resonance imaging (MRI), showing an enhanced ventral mass at the C5 level. (b) Initial axial Gd MRI showing a continuous intra- (black arrowhead) and extramedullary mass (white arrowhead). (c) Sagittal T2-weighted MRI showing hyperintensity from the medulla oblongata to the mid-thoracic spinal cord. (d) Contrast-enhanced computed tomography (CT) showing an enhanced cervical spinal cord mass and a right renal mass. (e) Lateral right vertebral angiogram showing the tumor stain involving the anterior spinal artery. (f) 3D rotational angiography of the right vertebral artery showing the stain of an intra- and extramedullary mass fed by the right radiculomeningeal artery (arrow) and anterior spinal artery (arrowhead). (g) Intraoperative exoscopic view showing an extramedullary hypervascular tumor ventrolateral to the cervical spinal cord and the nerve roots. (h) Intraoperative exoscopic view after midline myelotomy showing an intramedullary tumor. (i) The hematoxylin-eosin stain showing clear or eosinophilic cytoplasm due to glycogen and lipid accumulation, with well-defined cell borders and a nested or alveolar growth pattern surrounded by a delicate vascular network (a scale bar = 100 μm). (j) Immunohistochemical stain with PAX8, showing strong nuclear positivity, confirming their renal origin and distinguishing RCC from other neoplasms (a scale bar = 200 μm).

**Figure 2:**
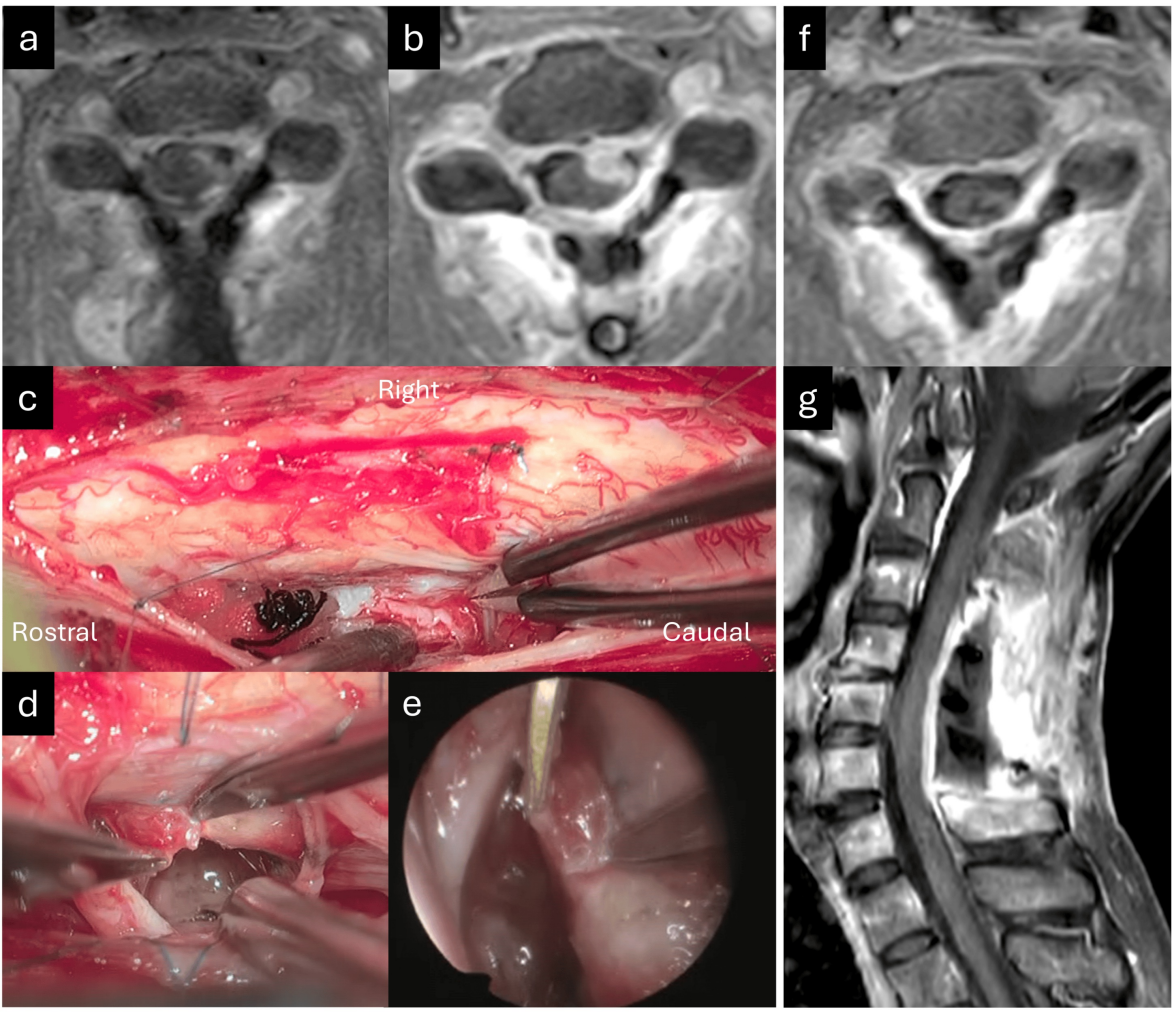
Preoperative, intraoperative, and postoperative findings at the second surgery (a) Axial contrast-enhanced gadolinium (Gd) magnetic resonance imaging (MRI) after the first surgery showing a small residual tumor ventrolateral to the cervical spinal cord. (b) Axial Gd MRI two months after the first surgery showing an enlarged residual tumor. (c) Intraoperative exoscopic view showing the recurrent tumor ventrolateral to the spinal cord. (d, e) Intraoperative exoscopic view and endoscopic view showing resection of the residual tumor adhesive to the ventral spinal cord with the simultaneous multi-angle view. (f, g) Postoperative axial and sagittal Gd MRI showing total removal.

In July 2024, A 73-year-old man experienced left neck and shoulder pain and left upper extremity weakness for two months. He had hypoesthesia, strength grade 4/5 in the left upper extremity and hyperreflexia of the upper and lower extremities without bladder dysfunction. Cervical Gd MRI revealed complex intra-and extramedullary lesions (Figures 1a-1b). T2-weighted MRI revealed hyperintensity from the medulla oblongata to the mid-thoracic spinal cord (Figure 1c). Because of the complex localization of the intra- and extramedullary tumors, a metastatic tumor was suspected. Contrast-enhanced computed tomography (CT) of the chest, abdomen, and pelvis was performed, which led to a diagnosis of RCC, without the detection of tiny acetabular bone metastasis in the first examination (Figure 1d). Gd brain and whole spine MRI revealed no other lesions. Digital subtraction angiography revealed that the right radiculomedullary artery fed the intra- and extramedullary tumors, the anterior spinal artery fed the intramedullary tumor (Figures 1e-1f), and the left radiculomedullary artery fed the extramedullary artery. The diagnosis was ISCM from RCC that broke the pia mater and extended to the left ventral extramedullary space, involving the anterior spinal artery. Preoperative endovascular embolization of the hypervascularized tumors was considered but was deemed dangerous because of the associated anterior spinal artery. The metastatectomy was prioritized over the nephrectomy due to the symptomatic and progressive nature of the spinal cord tumor, which posed an immediate risk of neurological compromise. Tumor excision was performed using C4-6 laminoplasty. First, the extramedullary tumor (Figure 1g) was resected after extirpation of the feeding artery from the left radiculomeningeal artery; however, the ventral side of the tumor could not be observed with an exoscope because of swelling of the spinal cord compressed by the intramedullary tumor. Second, the intramedullary tumor was removed (Figure 1h) via posterior midline myelotomy. Although discriminating the tumor from the normal spinal cord was relatively easy, the extramedullary tumor ventral to the anterior spinal artery was not removed to avoid neurological deterioration. Histopathological examination revealed metastatic clear cell RCC with less than 5% of the MIB-1 staining index and positive immunochemical staining for MDM2, PAX8, CD10, and CK AE1/AE3 (Figures 1i-1j). Postoperative Gd MRI showed a small residual tumor ventral to the spinal cord, which was intentionally left intraoperatively (Figure 2a). Postoperatively, the patient exhibited hypoesthesia and strength grade 3/5 in the left upper and lower extremities. These symptoms improved to the preoperative level following one month of rehabilitation. The nephrectomy was performed at the Department of Urology approximately one month after the first surgery for ISCM. The residual tumor rapidly increased for only two months after the resection of ISCM (Figure 2b). Reoperation of the extramedullary tumor was performed using an exoscope (Figures 2c-2d) and an endoscope (Figure 2e) to observe the ventral side of the spinal cord, achieving total resection of the tumor. The patient underwent radiotherapy to the C3-6 levels, receiving a total dose of 30 Gy delivered in 10 fractions, followed by chemotherapy with pembrolizumab. Following radiotherapy and chemotherapy, the patient reported pain relief, although the patient remained hypoesthesia and motor weakness (strength grade 4/5) in the left upper extremity, representing the same preoperative state. Postoperative cervical Gd MRI demonstrated no recurrence for four months (Figure 2f). Additionally, postoperative contrast-enhanced CT of the chest, abdomen, and pelvis revealed no other metastases, except for progression of the acetabular bone metastasis.

## Discussion

Only 36 cases of ISCM from RCC have been reported in the literature [1,2]. Most patients have systemic metastasis at the time of diagnosis [6]; nevertheless, ISCM can be the first manifestation of occult RCC [5]. There are five reported cases of ISCM as the solitary tumor without systematic metastasis [4-6,8,9] but with a history of RCC. Nine of 32 patients were simultaneously diagnosed with RCC and ISCM [1], as in the present case. Metastatic tumors should always be considered when intramedullary spinal cord tumors are detected on MRI. Positron emission tomography is recommended to diagnose ISCM, especially that of renal or lung origin [5,6].

The mean overall survival of patients with ISCM due to RCC is 13.15 months [1]. Neurological deterioration is exacerbated within several weeks, unlike primary intramedullary tumors, which typically progress slowly [5]. As RCC is radioresistant and chemotherapy is not feasible because of the blood-brain barrier [5], surgery is the first line of treatment for patients with neurological deficits caused by ISCM [1,5]. In a review of the literature [1], 66% of patients underwent surgical resection, 11 patients (58%) improved, seven patients (37%) were stable, and one patient (5%) deteriorated. Even if the tumor is resected totally, recurrent ISCM has been reported in the same location in the following months [6]; therefore, close follow-up with MRI is important. In the present case, the residual tumor showed progressive enlargement for only two months. Preoperative transarterial embolization is an option because RCC is a highly vascularized tumor [4]. In this case, selective embolization of the feeders associated with the anterior spinal artery would have been difficult, because even a small infarction of the spinal cord can lead to neurological deterioration.

Radiotherapy is an indispensable modality in the management of ISCM, particularly for patients who are not surgical candidates or have residual disease. Its primary objectives include providing symptomatic relief, arresting tumor progression, and preserving neurological function. Standard regimens encompass conventional fractionated radiotherapy (30 Gy in 10 fractions) and stereotactic body radiotherapy (SBRT), delivering 25-36 Gy in 5-6 fractions [10,11]. SBRT demonstrated exceptional efficacy in achieving high local control rates with minimal toxicity. Tonneau et al. reported that a median dose of 30 Gy in six fractions resulted in clinical stabilization or improvement in all patients, while Nishimura et al. highlighted that 30 Gy in 10 fractions preserved ambulation in 100% of ambulatory patients [10,11]. Despite concerns about the inherent radioresistance of tumors such as RCC, SBRT’s precision facilitates the delivery of ablative doses to the tumor while sparing adjacent spinal cord tissue. Radiation-induced myelopathy, a historically feared complication, is now rare with modern techniques adhering to strict dose constraints [1,10]. In the present case, the patient received a total dose of 30 Gy in 10 fractions to the C3-6 levels, resulting in stable neurological function post-treatment. This outcome underscores the safety and efficacy of this approach. While current evidence positions radiotherapy as an essential component of ISCM management, further investigations are warranted to refine fractionation protocols and identify optimal candidates for treatment [10,11].

Exophytic intramedullary spinal cord tumors are rare. There have been reports of exophytic intramedullary spinal cord tumors of glioblastoma [12,13], ependymoma [14], solitary fibrous tumor (SFT) [15,16], mature teratoma [17], Ewing sarcoma [18], and metastatic breast cancer [19]. This is the first reported case of exophytic ISCM from RCC. Dorsal extensions have been reported in almost all cases, while ventral extensions to the spinal cord are rare [13,16]. In dorsal exophytic tumors, the dorsal approach is easier and does not damage the normal spinal cord. In contrast, in ventral exophytic tumors, the exit zone is difficult to observe using the posterior approach, and the tumor may involve the anterior spinal artery. In the simple posterior approach, damage to the spinal cord and vasculature can induce severe neurological deterioration. However, the anterior approach is invasive to the vertebral column. Perrini et al. reported on the microsurgical resection of a cervical intradural juxtamedullary SFT with ventral extension. This study emphasizes the technical difficulties of firm tumor adhesion to the spinal cord and the absence of a clear arachnoid plane [16]. Exoscopic-endoscopic techniques are useful for lesions ventral to the spinal cord, especially to confirm residual tumors without damaging the spinal cord [7]. In contrast to a surgical microscope, an endoscopic view can be observed picture by picture using an exoscopic view, making it possible to perform the surgery at multiple angles. In the present case, the residual tumor adhering to the ventral cervical cord was safely resected, and all structures were identified.

## Conclusions

This report highlights a rare case of ventrally exophytic ISCM from RCC in the cervical spine. After initial resection using an exoscope, the residual tumor rapidly increased within two months, necessitating a second surgery after the nephrectomy. The use of combined exoscopic-endoscopic techniques enabled safe and complete resection of the ventral lesion while minimizing neurological risks.

This case emphasizes the importance of advanced minimally invasive surgical techniques for managing complex spinal lesions and the need for close postoperative monitoring to address rapid tumor progression. It also underscores the value of multidisciplinary strategies, including radiotherapy and systemic therapy, in optimizing outcomes for aggressive ISCMs.
